# Medical Opinion and Movement

**Published:** 1910-09-24

**Authors:** 


### Medical Opinion and Movement.

### Madelung's Deformity.

Two fresh cases of the somewhat rare condition known as Madelung's deformity are reported in the *Annals of Surgery* by Dr. A. C. Stokes. The condition is a dislocation forwards of the carpus, arising spontaneously; the degree of the dislocation is usually greater on the ulnar than on the radial side, and the symptoms are nearly always noticed first in persons under eighteen years of age. The patients are mostly of the lower classes, and many of them show marked evidence of rickets. There is a dystrophy and atrophy in the carpal bones, cartilages, and epiphyses; and the diaphysis of the radius is curved anteriorly. Heredity, female sex, and occupations throwing undue strain on the wrists are factors disposing to the disease. When the deformity is very great osteotomy may be justifiable; but it is indicated for its cosmetic effects only, and even for them it is often unsuccessful. Plaster casts, splints, etc., are useless. After the age of about twenty-five actual pain disappears, though the deformity, of course, remains; palliative treatment is therefore alone indicated up to that age. Counter-irritation, rest, and overfeeding are advised. Osteotomy is best postponed until symptoms have subsided. The prognosis as regards life is good; but for many years there may be some discomfort after exercise of the wrists.

### Heredity and Physical Defects.

Some extremely interesting tables showing the hereditary transmission of a tendency to some particular defect are published in the Report of the Medical Officer (Education) of the County of London for 1909. Lamellar cataract is especially prominent in this respect, and nine families have been followed up by Mr. Harman, in which the appearance of this trouble is extraordinarily persistent in successive generations. Thus in one family cataract was traced through five generations, and out of fifty-five individuals who survived infancy, nineteen were thus affected. In another family twelve out of twenty-seven are cataractous. Deafness, defective vitality and mentality, and insanity are also not very infrequent in such families. One case is of especial interest. Of fifteen cousins only three were cataractous; but one of these three married one of the unaffected cousins, and of their nine children five were certainly cataractous and two more may have been. The returns also show a more unusual defect handed on by heredity. A little girl suffered a spontaneous fracture of the left thigh in crossing her knees while sitting in a chair. It was then ascertained that her grandmother had broken her thigh seven times, an uncle once, and an aunt twice. Of the child's eleven cousins, one has had eleven fractures, one has twice had a broken thigh and five other fractures, and one twice a broken leg. The patient herself is now in a school for physically defectives.

### Phosphate-Uric Acid Ratio in Diagnosis.

Dr. Elsie Royle has recently been investigating various diseases from the point of view of their influence upon the ratio of phosphates to uric acid in the urine, and she has come to a provisional conclusion which may, if it is confirmed, be of the very greatest value in the diagnosis of the existence of malignant disease in cases in which, from the symptoms and signs, there is doubt. In estimating the ratio it is, of course, important to have the different patients upon such a diet that the amount of purin bases and of phosphates respectively in the food are either constant or actually known. It is found that in cases of cancer the uric acid in the urine is usually higher than it is in healthy patients or in patients suffering from non-malignant ailments under similar conditions; that the output of phosphates in the urine in the majority of cases of cancer is decreased when compared with the amounts passed by other individuals; and, further, that the ratio of $\frac{\text{phosphates}}{\text{uric acid}}$ is almost invariably reduced in malignant disease, and far more than it is under other circumstances. Upon a fixed diet, referred to by Dr. Royle as common diet, the kind of ratio found in non-malignant cases was anything from 4 to 10, whilst in many cases of malignant disease, whether carcinoma or sarcoma, and apparently no matter where the growth was situated, it would fall to anything from 3 down even to so low as 0.6. The figures, for instance, for four cases of gastric ulcer were 5.34, 6.42, 8.3, and 4.1; the corresponding figures for carcinoma of the stomach and œsophagus in individual cases were 2.22, 1.88, and 1.94. If differences of this kind were even usual without being absolutely universal, the value of the method in distinguishing between malignant and non-malignant diseases would clearly be very great. It would seem that any ratio below 4 is suggestive, especially if it remains low on several occasions, and any ratio below 3 is more than suggestive of malignant disease provided any of the blood diseases can be excluded on other grounds.

### Giant Cells in Syphilitic Lesions.

Giant cell systems are so prominent a feature of tuberculous lesions that it is supposed by many that their occurrence in microscopical section is almost conclusive evidence that the changes are tuberculous. It is true that text-books mention that giant cells are also found in other forms of chronic inflammation, especially in the granulomata which include, beside tubercle, syphilis, actinomycosis, and leprosy. It is noteworthy that these giant cells in syphilitic lesions are much more common than might be supposed, and this indicates the necessity for having sections stained bacteriologically when there is any doubt at all, in order to demonstrate the presence of tubercle bacilli within or near the giant cells before the latter are taken to be proof positive of tubercle. In a recent paper by Nicholas and Favre in the *Annales des Maladies Vénériennes*, this fact is brought out with particular clearness in connection with lesions of the skin. In twenty-four out of twenty-five cases of nodular secondary and tertiary syphilitic lesions there were found giant cells, epithelioid cells, and follicles in every respect identical with those of the most characteristic tuberculous lesions, so that even the most distinguished histologists, when shown a series of sections, some of which were obtained from averred syphilitic lesions, proved so by the history, clinical characters, negative results of cultures and inoculation of guinea-pigs, and rapid recovery under mercury or iodide, and others from lesions proved to be tuberculous, failed to distinguish one from the other.

### Vaccine Therapy in Bacillus Coli Infection.

Billings (*American Journal of Medical Science*), from a study of bacteriuria during the last five years, urges the value of coli-vaccine therapy in those cases that are due to the bacillus coli communis, comprising, as they do, more than 50 per cent. of all cases of bacteriuria. Coli bacilluria may be unattended by symptoms, or it may give rise to dysuria, and, when associated with definite morbid changes in the urinary tract, may be either causative or closely related to the accompanying disease process. When bacillus coli infection was associated with tuberculosis of the urinary tract the dysuria and fever were almost entirely relieved by the disappearance of the coli infection after autogenous vaccination. Improvement may occur under treatment in all cases; but if there is stagnation anywhere in the urinary tract a complete recovery can hardly be looked for unless this can be cured by surgical interference or otherwise. Since the bacilli from different cases of bacilluria differ in size, luxuriance of growth, etc., autogenous vaccines are desirable, and cultures can easily be made from the urine, even after it has been sent considerable distances to a laboratory for that purpose. The vaccine may be made by heating the culture to 60° C. for thirty minutes, thereby killing the bacilli; and fresh suspensions should be used, as those more than a fortnight old may not give the same results. The first vaccination is usually made with 200 million bacilli, the subsequent dosage being increased until a decided local or general reaction occurs. The maximum dose was 1,000 millions, and with some patients it was found that smaller doses (five to a hundred millions) produced sufficient reaction for curative purposes without risking too great a reaction. Absolute rest, milk diet, and plenty of fluid accompanied the treatment, thereby reducing the risk of chill and making recovery more certain. The local reaction consists of redness, tenderness, and swelling over an area two inches square at the site of injection, commencing two hours after injection, reaching a maximum in twelve to eighteen hours, and gradually disappearing in forty-eight to seventy-two hours. The general reaction of malaise, aching, headache, and fever usually occurs in from two to twelve hours.

### Rectal Myoma.

Intestinal fibromyomata are rare, only about eighty cases having been reported of which but sixteen involved the rectum. Descoeudres, in *La Revue de Gynécologie et de Chirurgie Abdominale*, describes a case in which such a tumour developed backwards under the skin of the buttocks, attached by a fibrous pedicle to the rectal wall. The tumour was so large as to hang down and cause inconvenience in walking, but it was painless on pressure. In commenting on the case the author points out that these growths are often congenital, embryonic tissue developing as the result of irritation. The muscular coat of the intestine suffers in consequence. They cause constipation, hæmorrhage and œdema of the legs from pressure on the veins. The only rational treatment is removal, since apart from the possibility of their becoming malignantly degenerated they tend if left to produce a cachectic condition from intestinal obstruction and repeated hæmorrhage. They may occur also internal to the gut, as polypi.

### Priapism Secondary to Appendicitis.

Dr. Felix Rosenthal, in the *Berliner Klin. Woch.*, reports a case of post-appendicular priapism in a man of forty-two. The patient had suffered from diffuse intra-abdominal pain, later localised in the right iliac fossa, at which time a resistance could be felt in the ileo-cæcal region, which was tender to pressure. These signs, together with fever and a leucocytosis pointed to acute appendicitis, a diagnosis which was confirmed by rectal examination which revealed a tender resistant swelling in the region of the prostate gland. Four days after the first symptoms the penis was seen to be in a position of complete and permanent erection. The corpora cavernosa alone were affected, being hard, equally distended, and somewhat tender. The glans and corpus spongiosum were soft. There was slight pain on micturition. The appendicitis was found at operation to be of the pelvic type. A tumour limited to the right side, near the bladder, pressed upon the rectum on that side above the prostate. It is probable that infection of the deeper veins of the penis from the septic focus had brought about a spreading thrombosis of those of the corpora cavernosa. Puncture of these latter, moreover, yielded a fluid rich in bacillus coli. The patient was cured without further intervention, though twelve days after leaving hospital some traces of induration of the corpora cavernosa still remained. The author thinks that the case is probably unique.

### Sebaceous Cysts.

The usual method of removing a sebaceous cyst is to make an incision of the skin over the cyst and carefully dissect it out, freeing the sac wall from the adhesions to the adjacent tissues. Dr. H. Freeth, of Llangollen, proposes a somewhat different method of procedure, for which he claims several advantages. He makes the incision into the skin at the side of the cyst and parallel with it, and then insinuates a blunt dissector; with which he separates the adhesions, at first on the superficial side, where the wall of the cyst is usually thin, and then on the deeper side, so that the tumour is completely isolated. By a little pressure the tumour can then be delivered similarly to the manner of extracting a cataract. The advantages claimed are that the incision is smaller and is made in healthy skin and consequently heals more rapidly and leaves a better scar. The operation requires a minimum of anæsthetic, it being sufficient to make a hypodermic injection in the line of incision. Although the skin over the cyst may be much stretched and altered, it quickly retracts and resumes a normal aspect. The author has removed in this way cysts as large as a hen's egg.

### Hæmoptysis and Blood Pressure.

It has already been demonstrated by Neumann, Hensen, and others that in tuberculous subjects affected with pulmonary disease, the blood pressure is usually considerably below normal, but that hæmoptysis is always preceded and accompanied by a rise in blood pressure. Dr. Müller reports similar results from his observations in the *Wiener Medizinische Wochenschrift*, and he further points out the practical significance of these facts and the clinical utility to which they may be put. By systematic blood-pressure observations on these patients, any elevation in the blood-pressure is noted and precautions at once taken against the occurrence of hæmorrhage. The measures suggested by the author are complete rest in bed and the administration of morphia and digitalis. The author claims to have succeeded in many cases in preventing the occurrence of hæmorrhage by these precautionary measures. He also gives an account of two cases in which his advice, in consequence of a raised blood-pressure, to rest in bed was not attended to, and hæmoptysis, in one case of a severe nature, ensued. The author records cases in which hæmorrhage has occurred after a rise of only 10 mm. in the blood-pressure. Other cases have shown a rise of 20 mm. or more, but the blood-pressure may begin to rise several weeks before the occurrence of a hæmorrhage, so that by regular observations it is possible to anticipate the hæmorrhage sufficiently early to carry out the preventive treatment, and it should be continued till the blood-pressure has returned again to its original level.

### Subcutaneous Injection of Oxygen.

In a contribution to *Le Progrès Médical* Dr. F. Ramond describes his method of treating conditions of asphyxia, toxic or otherwise, by subcutaneous injections of oxygen. In cases of asphyxia from some mechanical obstruction in the air passage, the treatment can only give but doubtful and temporary relief, the only proper remedy being removal of the obstruction; but it may serve to ward off imminent danger in the meanwhile. The injections are of greater service in the various conditions of toxic asphyxia such as uræmia, diabetic coma, and in poisoning by carbonic acid, carbon monoxide, ether, and chloroform. Of even greater benefit is the treatment when mechanical and toxic factors are combined—such conditions as bronchitis with emphysema or cardiac affection and pneumonia. The oxygen injections not only supply the needed aeration to the tissues, but stimulate the organism, and by oxidising the toxins render them innocuous. The technique is quite simple. The skin over the abdomen or thigh is disinfected and a syringe needle passed through the skin into the subcutaneous tissue. By attaching a syringe and aspirating, it can be ascertained if the needle has penetrated a vein. In such a case the syringe will fill with blood, and the needle must be withdrawn and a second puncture made. The needle is then connected with the supply of oxygen by a rubber tube and the oxygen allowed to flow slowly in. A cottonwool plug may be inserted in the neck of the needle to filter the oxygen from any solid particles. About 1½ litre may be injected, and the operation may be repeated several times a day. According to the author, the patient frequently obtains so much relief that he clamours for a constant renewal of the treatment. The author has also tried intra-rectal injections, but the beneficial results are obtained more slowly. He thinks the two methods might be combined.

### The Ultra-violet Rays.

Some interesting research work on the ultra-violet rays of light is recorded in the *Lyon Médical* by Dr. H. Bordier. By a special apparatus he has studied the penetrating powers of these rays in relation to all manner of substances and solutions, including the fluids and tissues of the human organism. He finds that alcohol and chloroform are more opaque to these rays than water, and in regard to colloidal substances, such as gum arabic, gelatine, and albumin, their opacity is inversely proportional to the amount of water they contain. Metals in the colloidal state completely arrest the ultra-violet rays. The organic fluids vary in their transparency. Cerebrospinal fluid is a little more opaque than water, pleuritic fluid more so, and milk and urine completely arrest these rays. In regard to the tissues of the eye, vitreous humour is as transparent as water. The cornea arrests a large proportion of the rays, thus protecting the deeper parts of the eye. The crystalline lens is completely opaque to these rays. It becomes fluorescent under the influence of these rays, and in fulfilling the duty of protecting the retina in this way the author suggests that it may itself suffer from their action, resulting in the early production of cataract, as in the case of glass-makers and founders. Eyes operated upon for cataract and in consequence deprived of their natural powers of defence should be specially protected against the action of the ultra-violet rays. The author points out further that such lights as the arc lamp, the Auer light, and the mercury vapour light should only be used for large spaces. On the other hand, the work table or desk should be lighted by the oil lamp or electric bulb, which are very feeble in ultra-violet rays. He makes the further interesting observations that the dust and smoke in the atmosphere of towns occludes these rays, and hence sunstroke is much rarer here than in the country and on the mountains. On the other hand, he suggests that the tonic effects of the country air are due to the same cause.

